# T2-weighted, apparent diffusion coefficient and ^18^F-FDG PET histogram analysis of rectal cancer after preoperative chemoradiotherapy

**DOI:** 10.1007/s10151-021-02440-9

**Published:** 2021-04-01

**Authors:** F. Crimì, R. Stramare, G. Spolverato, V. Aldegheri, A. Barison, L. D’Alimonte, Q. R. Bao, A. Spimpolo, L. Albertoni, D. Cecchin, C. Campi, E. Quaia, S. Pucciarelli, P. Zucchetta

**Affiliations:** 1 Institute of Radiology, Department of Medicine (DIMED), University Hospital of Padova, Padova, Italy; 2 Clinica Chirurgica I, Department of Surgical, Oncological and Gastroenterological Sciences (DiSCOG), University Hospital of Padova, Via Nicolò Giustiniani 2, 35128 Padova, Italy; 3 Nuclear Medicine Unit, Department of Medicine (DIMED), University Hospital of Padova, Padova, Italy; 4 Surgical Pathology and Cytopathology Unit, Department of Medicine (DIMED), University Hospital of Padova, Padova, Italy; 5 Department of Mathematics “Tullio Levi-Civita”, University of Padova, Padova, Italy

**Keywords:** Rectal cancer, Preoperative chemoradiotherapy, Magnetic resonance imaging (MRI), Positron emission tomography (PET)

## Abstract

**Background:**

The aim of our study was to investigate the correlation among T2-weighted (T2w) images, apparent diffusion coefficient (ADC) maps, ^18^F-fluorodeoxyglucose (^18^F-FDG) positron emission tomography (PET) images, histogram analysis and the pathological response in locally advanced rectal cancer (LARC) after preoperative chemoradiotherapy (pCRT).

**Methods:**

Patients with LARC were prospectively enrolled between February 2015 and August 2018 and underwent PET/magnetic resonance imaging (MRI). MRI included T2w and diffusion-weighted imaging (DWI)-sequences. ADC maps and PET images were matched to the T2w images. Voxel-based standardized uptake values (SUVs,) ADC and T2w-signal-intensity values were collected from the volumes of interest (VOIs) and mean, skewness and kurtosis were calculated. Spearman’s correlation coefficient was applied to evaluate the correlation among the variables and tumor regression grade (TRG), T stage, N stage and fibrosis.

**Results:**

Twenty-two patients with biopsy-proven LARC in the low or mid rectum were enrolled [17 males, mean age was 69 years (range 49–85 years)]. Seven patients experienced complete regression (TRG1). A significant positive correlation was found between SUV mean values (*ρ* = 0.480; *p* = 0.037) and TRG. No other significant correlations were found.

**Conclusions:**

Histogram analysis of SUV values is a predictor of TRG in LARC.

## Introduction

Colorectal cancer is the third most common cause of tumor-related death in women and the fourth in men and rectal cancer accounts for about 30% of all colorectal cancers [1]. The standard of care for locally advanced rectal cancer (LARC) is preoperative chemoradiotherapy (pCRT) followed by total mesorectal excision (TME) [2].

After pCRT approximately 15–20% of these patients have a complete pathological response (pCR) with no tumor remnant at the histopathological examination [3, 4]. For this reason, after pCRT a rectal-sparing approach such as transanal local excision (LE) or a watch-and-wait approach could be considered instead of total mesorectal excision (TME) surgery in patients with major or complete clinical response (i.e. without residual tumor on imaging and endoscopy), as supported by a growing body of evidence [3, 5, 6]. These approaches appear safe only for a highly selected subset of patients, therefore strict selection criteria and close follow-up, including endoscopy and magnetic resonance imaging (MRI) should be used [4].

Moreover, the definition of complete clinical response, especially with imaging, and the correlation between it and the histopathological features is challenging [7]. MRI is routinely adopted to evaluate the response to pCRT, especially with diffusion-weighted imaging (DWI) sequences [8]. Different dimensional criteria have been used to evaluate the reduction of tumor size after pCRT, such as the response evaluation criteria in solid tumors (RECIST), one-dimensional and volumetric criteria, with the volumetric measurement showing a better reproducibility [8–11]. Semi-quantitative grading of the fibrosis within the primary lesion (low signal intensity on T2-weighted sequences) has been proposed to evaluate the response to pCRT, in analogy with the pathologic tumor regression grade (TRG). However, low agreement with histopathological TRG and low specificity in the identification of the pCR of 62.8% were found [12]. Recently, automatic quantification of the T2-weighted signal intensity inside the tumor volume has been tested, with encouraging results in differentiating good and poor responders to pCRT with higher sensitivity and specificity (78.26% and 97.62%, respectively) [13].

DWI sequences have improved the accuracy of tumor staging compared to classical MRI, which relies exclusively on morphological sequences [14]. The combination of MRI morphological T2-weighted features and findings from DWI sequences have been used to identify specific patterns of fibrotic tumor response to pCRT, showing a sensitivity of 94% and a specificity of 77% for the identification of pCR [15]. The role of quantitative evaluation of apparent diffusion coefficient (ADC) maps in response to chemoradiation is still debated [8], even if various studies have generally confirmed an increase in ADC in post-pCRT MRI scans when compared with preoperative scans, due to tissue necrosis inside the tumor [16, 17]. Volumetric analysis of ADC maps has been proposed but did not show significant differences from the evaluation of the more straightforward mean ADC value [13]. In a recently published paper, only a correlation between post pCRT skewness and TRG was revealed [18]. Moreover, no significant correlations have been reported between histogram analysis of dynamic contrast-enhanced MRI with TRG [19].

^18^F-fluorodeoxyglucose positron emission tomography/computed tomography scan ( ^18^F-FDG PET/CT) is another useful tool, even if not routinely recommended in patients with LARC, that in a recent meta-analysis showed a sensitivity of 73% and a specificity of 77% for the identification of responders to pCRT [20]. ^18^F-FDG PET/MRI has been proposed as a new technique to stage and restage rectal cancer [21]. In a recent study on pelvis malignancies, no significant differences in ADC and standardized uptake value ( SUV) metrics have been shown in PET/MRI, suggesting that a combined SUV + ADC index would be useful to evaluate the pathology [22].

The combination of morphological and functional information of MRI-DWI with the metabolic information of PET could potentially allow a precise evaluation of tumor response to pCRT.

We aimed to perform a histogram analysis to investigate the correlation between T2-weighted (T2w) signal intensity, ADC, and SUV values in the volume of interest (VOI) of the primary lesion and histopathological TRG, T stage, N stage and fibrosis percentage in patients affected by LARC after pCRT.

## Materials and methods

### Patients selection

A prospective study following the Helsinki declaration was approved by the local ethics committee. Between February 2015 and August 2018 patients with LARC were consecutively enrolled. All patients gave signed informed consent. Inclusion criteria were: (i) biopsy-proven low-mid rectal cancer (less than 12 cm from the anal verge); (ii) pCRT with iperfractioned radiotherapy (1.8 Gy/day with a total of 50.4 Gy) and concomitant chemotherapy with venous infusion based on 5-fluorouracil or oral capecitabine; (iii) restaging with ^18^F-FDG PET/MRI at least 5 weeks after the completion of chemoradiation; (iv) surgical intervention 6–8 weeks after the conclusion of pCRT. Exclusion criteria were: (i) high rectal tumor (> 12 cm from anal verge); (ii) stage cTNM I or IV at baseline; (iii) restaging performed without PET/MRI after pCRT; (iv) lack of informed consent. In the case of major or complete clinical response to pCRT, patients were asked to undergo local excision (LE) instead of classical TME. Complete clinical response was defined as: (a) no palpable mass at the digital rectal examination; (b) no evidence of residual tumor and a white scar at endoscopy; (c) substantial downsizing at MRI with a normal rectal wall or residual fibrosis/residual wall thickening because of edema and no suspicious lymph nodes. Clinical response was defined as major when one or two of the complete response criteria were missing. In these cases, the following were considered as indicative of a clinical response: (a) small superficial soft irregularity or no palpable mass at the digital rectal examination; (b) small mucosal irregularity or superficial ulcer no more than 2 cm in diameter at endoscopy; (c) obvious downstaging of the lesion at MRI with residual fibrosis but heterogeneous or irregular aspect or obvious downstaging of lymph nodes but remaining nodes ≥ 5 mm without malignant enhancement pattern [4].

### Histopathology examination

A dedicated pathologist examined all the surgical specimens and reported the histopathological findings according to the 8th edition of the American Joint Committee on Cancer classification [23]. Post-chemoradiotherapy histopathological T staging (ypT) and N staging (ypN), when TME was feasible, were reported. In patients that underwent LE, the negative follow-up for nodal metastases was used as a reference standard to replace the histopathological evaluation of local lymph nodes.

The TRG of the primary lesion was reported according to the Mandard classification: TRG 1 complete regression with fibrosis and absence of residual cancer cells; TRG 2 presence or rare residual cancer cells; TRG 3 presence of residual tumor with predominantly fibrosis; TRG 4 residual cancer outgrowing fibrosis; TRG 5 no regressive change of the tumor [24]. In each histopathological specimen, the pathologist quantified fibrosis in a semi-quantitative way (from 0 to 4) to verify if the amount of fibrosis may mislead the evaluation of neoplastic regression on imaging.

### Imaging techniques

Restaging ^18^F-FDG PET/MRI was performed in all patients, using endo-rectal ultrasound gel positioned before the acquisition. We used an integrated 3T PET/MRI (Biograph mMR, Siemens Healthineers, Germany) and the MRI protocol including (i) pelvic axial-oblique T2w turbo spin-echo (TSE) with slice thickness 3 mm, echo time (TE) 123 ms and repetition time (TR) 4540; (ii) axial DWI sequences with slice thickness 5 mm, TE 72 ms, TR 5100 ms, and b-values 50–1000 s/mm^2^. PET images were acquired simultaneously, after around 60 min from the tracer injection (3 Mbq/kg of ^18^F-FDG), with a bed duration of 20 min; attenuation correction maps were generated using a VIBE-Dixon sequence.

### Image analysis

For image analysis, the software PMOD (PMOD Technologies LLC, Switzerland) on a dedicated workstation was used. ADC maps and PET images of the pelvis were resliced and reoriented to match with the T2w axial-oblique images perfectly.

**Fig. 1:**
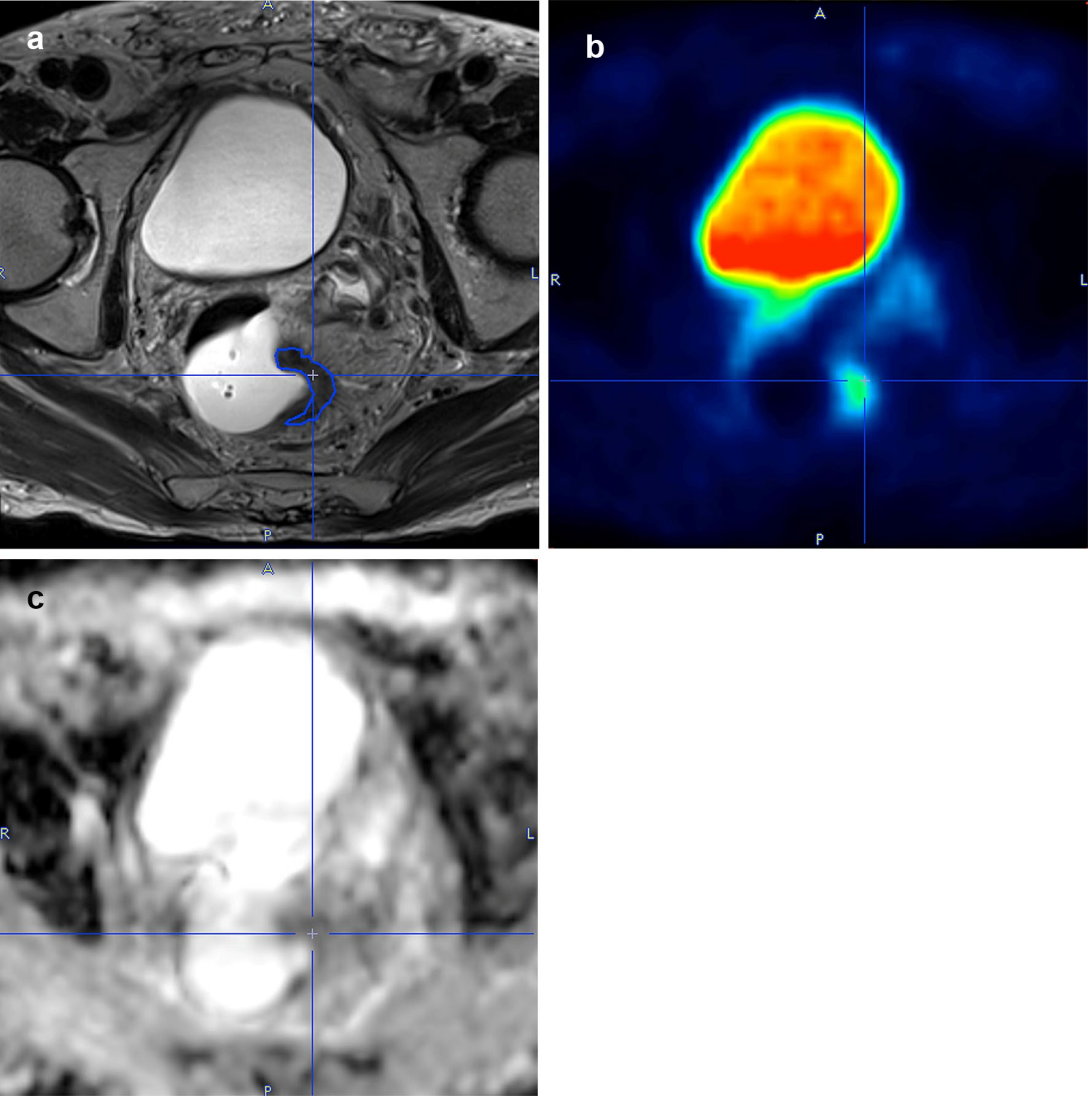
Delineation of the volume of interest with PMOD in a mid-rectal cancer on T2w axial-oblique images (**a**) and the corresponding PET images (**b**) and ADC maps (**c**). *T2w* T2-weighted; *PET* positron emission tomography; *ADC* apparent diffusion coefficient

Two radiologists consensually delimited a region of interest (ROI) around the margins of the primary lesion in each slice of the T2w images (Fig. 1a), including rectal cancer, thus obtaining a volume of interest (VOI). Each ROI was then copied on the corresponding PET (Fig. 1b) and ADC slices (Fig. 1c). The dimensions of each voxel in the VOI were 0.75 × 0.75x3.00 mm; voxel-based SUV, ADC, and T2w signal intensity values were extracted from the volume of the tumor. T2w signal intensity values were normalized in each patient based on the mean signal intensity of a 10 mm diameter spherical VOI in the subcutaneous fat.

### Statistical analysis

Mean, skewness and kurtosis were calculated from VOIs of each dataset (T2w and PET images and ADC maps). Spearman’s correlation coefficient was applied to evaluate the correlation among the variables and TRG, ypT, ypN, and the semi-quantitative amount of the fibrosis. The level of significance was set at *p* ≤ 0.05. R software was used to perform the statistical analysis [25].

## Results

Overall, 22 patients with biopsy-proven LARC in the low or mid rectum were enrolled, 17 (77.3%) were males and 5 (22.7%) females. The mean age was 69 years (range 49–85 years). Seventeen patients (77.3%) underwent TME while 5 patients (22.7%) with major or complete clinical response to pCRT underwent LE, as part of a prospective observational trial (NCT0271081) [5]. Patients treated with LE underwent strict endoscopic and MRI monitoring (every 3 months after surgery) to exclude local or regional lymph node recurrences (at least 1 year of negative follow-up). Overall, 7 patients had a TRG 1, 1 TRG 2, 7 TRG 3, 6 TRG 4 and 1 TRG 5. The majority of patients were either T0 (*n* = 7, 31.8%) or T3 (*n* = 8, 36.4%). At the final pathology 10 patients were node negative, while 7 had metastatic nodes. Most of patient had grade 1 (*n* = 11, 50.0%) and 2 fibrosis (*n* = 7, 31.8%) (Table 1).

**Table 1:** Histopathological data of patients

Case	Surgery	TRG	ypT	ypN	Fibrosis
1	TME	1	0	0	1
2	TME	1	0	0	1
3	TME	1	0	0	2
4	TME	1	0	1a	2
5	TME	3	2	0	2
6	TME	3	3	1b	1
7	TME	3	3	2a	0
8	TME	3	3	2b	2
9	TME	3	3	0	2
10	TME	3	4b	0	1
11	TME	3	4b	0	2
12	TME	4	2	0	0
13	TME	4	3	0	0
14	TME	4	3	2a	0
15	TME	4	3	2b	1
16	TME	4	4b	1b	2
17	TME	5	3	0	1
18	LE	1	0	X	1
19	LE	1	0	X	1
20	LE	1	0	X	1
21	LE	2	1	X	1
22	LE	4	1	X	1

*TME* total mesorectal excision; *LE* local excision; *TRG* tumor regression grade; *ypT* histopathological T stage after preoperative chemoradiotherapy; *ypN* histopathological N stage after preoperative chemoradiotherapy; *X* indeterminable, histological examination of the lymph nodes was not possible in patients operated with local excision, therefore N indeterminable was reported for these cases

### VOI analysis

The Spearman’s correlation coefficient test showed a statistically significant positive correlation only between the mean SUV of the lesions and TRG (*ρ* = 0.47; *p* = 0.03). Therefore, the higher SUV mean of the lesion was at PET examination, the higher TRG was found at histopathological examination. The residual neoplastic tissue is avid of 18F-FDG because of its high glycolytic metabolism, hence shows a higher SUV mean in PET images, while fibrous scar tissue does not show radiotracer uptake, having a lower SUV mean. A trend towards significance was found between ADC kurtosis and TRG (*ρ* = − 0.40; *p* = 0.06), between SUV kurtosis and TRG (*ρ* = − 0.40; *p* = 0.07) and between SUV kurtosis and N stage (*ρ* = − 0.39; *p* = 0.07) (Tables 2, 3, 4). The kurtosis of the ADC and SUV histogram distribution was negatively correlated with TRG, meaning that the more platykurtic were the correspondent distributions (i.e. a distribution shape with “thinner tails”) the higher was the TRG at histopathology and therefore the tumor remnant. In the same way, platykurtic distributions of SUV were related to the presence of nodal metastases.

**Table 2:** ADC, SUV and T2w signal intensity correlations with TRG

TRG	*p* value	*ρ*
ADC		
Mean	0.90	0.03
Skewness	0.13	0.33
Kurtosis	0.06	− 0.40
SUV		
Mean	0.03	0.47
Skewness	0.76	0.07
Kurtosis	0.07	− 0.40
T2w		
Mean	0.26	0.25
Skewness	0.95	− 0.01
Kurtosis	0.92	− 0.02

*TRG* tumor regression grade; *ADC* apparent diffusion coefficient; *SUV* standardized uptake value; *T2w* T2-weighted signal intensity value; *ρ* rho coefficient

**Table 3:** ADC, SUV and T2w signal intensity correlations with ypT stage

ypT stage	*p* value	*ρ*
ADC		
Mean	0.81	− 0.05
Skewness	0.85	0.04
Kurtosis	0.58	− 0.12
SUV		
Mean	0.74	− 0.07
Skewness	0.13	− 0.33
Kurtosis	0.73	0.08
T2w		
Mean	0.90	0.03
Skewness	0.99	− 0.01
Kurtosis	0.10	− 0.36

*ypT* histopathological T stage after preoperative chemoradiotherapy; *ADC* apparent diffusion coefficient; *SUV* standardized uptake value; *T2w* T2-weighted signal intensity value; *ρ* rho coefficient

**Table 4:** ADC, SUV and T2w signal intensity correlations with ypN stage

yN stage	*p* value	*ρ*
ADC		
Mean	0.52	− 0.15
Skewness	0.27	0.24
Kurtosis	0.89	− 0.03
SUV		
Mean	0.37	0.20
Skewness	0.71	0.08
Kurtosis	0.07	− 0.39
T2w		
Mean	0.40	0.19
Skewness	1.00	0.00
Kurtosis	0.99	0.01

*ypN* histopathological N stage after preoperative chemoradiotherapy; *ADC* apparent diffusion coefficient; *SUV* standardized uptake value; *T2w* T2-weighted signal intensity value; *ρ* rho coefficient

We did not find any other correlation among ADC, SUV, and T2W signal intensity mean, skewness and kurtosis and T stage, N stage, or fibrosis percentage (Tables 3, 4, 5). The results for fibrosis somehow underline how the fibrotic reaction within the whole specimen is not linked to a misleading evaluation of neoplastic regression on imaging because it showed different results from TRG.

**Table 5:** ADC, SUV and T2w signal intensity correlations with fibrosis

Fibrosis	*p* value	*ρ*
ADC		
Mean	0.86	0.04
Skewness	0.39	0.19
Kurtosis	0.20	0.28
SUV		
Mean	1.00	0.00
Skewness	0.19	− 0.29
Kurtosis	0.45	0.17
T2w		
Mean	0.57	− 0.13
Skewness	0.19	− 0.29
Kurtosis	0.57	− 0.13

*ADC* apparent diffusion coefficient; *SUV* standardized uptake value; *T2w* T2-weighted signal intensity value; *ρ* rho coefficient

## Discussion

Current pCRT regimens bring to a complete response of the tumor in a range between 8–24% of cases [4, 5], and recent studies showed that in patients with complete or major clinical response, a less aggressive approach could be performed with a LE or a “watch-and-wait” approach, avoiding major surgery and averted permanent colostomy without loss of oncological safety for at least 3 years [3, 26, 27]. In an observational study of 880 patients with complete clinical response undergoing a “watch-and-wait” approach, Van der Valk found that 88% of all cases of recurrence were diagnosed in the first 2 years of follow-up, 97% were intraluminal and distant metastases were diagnosed in 71 (8%) of 880 patients. Five-year overall survival was 85% (95% CI 80·9–87·7%), and 5-year disease-specific survival was 94% (91–96%) [28]. Therefore, the accuracy of restaging after pCRT is pivotal to guide the treatment plan correctly. Patients selection for nonoperative management relies mainly on restaging performed 6–8 weeks after the end of pCRT with endoscopy and biopsies to assess the tumor depth and MRI to characterize the nodes [29]. The international guidelines recommend MRI as the main imaging tool to restage patients with advanced rectal cancer after pCRT [30]. The current MRI imaging protocols with DWI allow to achieve a high accuracy for the identification of pCR in the primary lesion, especially with the qualitative evaluation of the DWI sequences [15]. Indeed, a recognized mean cut-off value for the identification of the pCR has not been established yet, even if a correlation between TRG and ADC has been widely demonstrated [8, 16, 17, 31]. The analysis has been performed mostly comparing the ADC before and after pCRT, with an increase in ADC corresponding to a response to treatment [31]. The quantitative evaluation of T2w images, and especially the texture analysis, recently proved to be an effective tool in restaging rectal cancer with an excellent concordance with the TRG [13, 32–34].

The reduction of metabolic parameters of the primary lesion due to pCRT has been demonstrated to correlate with pCR in PET studies [20]. In the present study, the only technique that showed a statistically significant correlation with TRG was ^18^F-FDG PET. In particular, the mean SUV values of the lesions were positively correlated with the TRG; therefore, a lower SUV mean value corresponded to a lower TRG, indicating a smaller residual tumor and so a better response to pCRT. This result was in accordance with the findings of the published literature on PET imaging, which showed a correlation between SUV mean values and TRG [20, 35–37]. Moreover, a trend toward significance with a negative correlation was detected between SUV kurtosis and TRG and SUV kurtosis and N stage. Nevertheless, these results should be confirmed in a larger cohort, potentially demonstrating that PET histogram analysis could be an interesting tool in the evaluation of rectal cancer after pCRT.

No significant correlation was found on the ADC histogram analysis, and there was only a trend toward significance for a negative correlation between ADC kurtosis and TRG. The applicability of ADC histogram analysis is still debated. Curvo-Semedo et al. reported a good ability of DWI sequences to quantify the tumor volume reduction, while the evaluation of pre and post pCRT ADC in a ROI showed a low accuracy for pCR identification [38]. On the contrary, Enkhbaatar and colleagues, using a volumetric analysis of the primary lesions, found a correlation among post-pCRT ADC skewness and percentage reduction of ADC with the response to therapy [18]. The differences from our study were the type of rectal distension (ultrasound gel in our study vs. barium of Enkhbaatar’s work) and lower statistical power for the lower number of patients examined.

In the T2w signal intensity histogram analysis, we did not demonstrate any statistically significant correlation with TRG, T stage, N stage. In the literature, the vast majority of the studies performed a comparison with both the pre-pCRT and post-pCRT MRI scans or only with the pre-treatment MRI images. It is probably necessary to have a baseline evaluation before chemo-radiation to correctly detect the therapy-induced fibrosis in the lesion [13, 32–34].

Only one other study, by Giannini and colleagues, compared MRI, PET, and DWI features in rectal cancer with TRG, using the texture analysis of ^18^F-FDG PET/CT and MRI before chemoradiation. They showed that a logistic regression model, including PET and T2w second-order parameters, is a good predictor of the complete pathological response of rectal cancer to pCRT (sensitivity and specificity of 86% and 83%) [32].

Our study has some limitations that should be considered when interpreting the results: first, the small sample size, mainly due to the complexity and the costs of the PET/MRI. Second, we did not use normalization of the signal intensity of the T2w images, even if the parameters used for the sequences were the same in all patients. Finally, the endorectal gel covering the luminal part of the tumor can have influenced, even if minimally, the values of ADC and T2w signal intensity extracted from the images.

## Conclusions

We demonstrated the feasibility of histogram analysis of PET/MRI imaging in rectal cancer after pCRT, and we found that some parameters, especially of PET images, can have a role as potential markers of tumor response to chemoradiation.
